# Examining Twitter Discourse on Electronic Cigarette and Tobacco Consumption During National Cancer Prevention Month in 2018: Topic Modeling and Geospatial Analysis

**DOI:** 10.2196/28042

**Published:** 2021-12-29

**Authors:** Jiahui Lu, Edmund W J Lee

**Affiliations:** 1 School of New Media and Communication Tianjin University Tianjin China; 2 Wee Kim Wee School of Communication and Information Nanyang Technological University Singapore Singapore

**Keywords:** electronic cigarette, smoking, lung cancer, Twitter, national cancer prevention month, policy, topic modeling, cessation, e-cigarette, cancer, social media, eHealth, cancer prevention, tweets, public health

## Abstract

**Background:**

Examining public perception of tobacco products is critical for effective tobacco policy making and public education outreach. While the link between traditional tobacco products and lung cancer is well established, it is not known how the public perceives the association between electronic cigarettes (e-cigarettes) and lung cancer. In addition, it is unclear how members of the public interact with official messages during cancer campaigns on tobacco consumption and lung cancer.

**Objective:**

In this study, we aimed to analyze e-cigarette and smoking tweets in the context of lung cancer during National Cancer Prevention Month in 2018 and examine how e-cigarette and traditional tobacco product discussions relate to implementation of tobacco control policies across different states in the United States.

**Methods:**

We mined tweets that contained the term “lung cancer” on Twitter from February to March 2018. The data set contained 13,946 publicly available tweets that occurred during National Cancer Prevention Month (February 2018), and 10,153 tweets that occurred during March 2018. E-cigarette–related and smoking-related tweets were retrieved, using topic modeling and geospatial analysis.

**Results:**

Debates on harmfulness (454/915, 49.7%), personal experiences (316/915, 34.5%), and e-cigarette risks (145/915, 15.8%) were the major themes of e-cigarette tweets related to lung cancer. Policy discussions (2251/3870, 58.1%), smoking risks (843/3870, 21.8%), and personal experiences (776/3870, 20.1%) were the major themes of smoking tweets related to lung cancer. Geospatial analysis showed that discussion on e-cigarette risks was positively correlated with the number of state-level smoke-free policies enacted for e-cigarettes. In particular, the number of indoor and on campus smoke-free policies was related to the number of tweets on e-cigarette risks (smoke-free indoor, *r*49=0.33, *P*=.02; smoke-free campus, *r*49=0.32, *P*=.02). The total number of e-cigarette policies was also positively related to the number of tweets on e-cigarette risks (*r*49=0.32, *P*=.02). In contrast, the number of smoking policies was not significantly associated with any of the smoking themes in the lung cancer discourse (*P*>.13).

**Conclusions:**

Though people recognized the importance of traditional tobacco control policies in reducing lung cancer incidences, their views on e-cigarette risks were divided, and discussions on the importance of e-cigarette policy control were missing from public discourse. Findings suggest the need for health organizations to continuously engage the public in discussions on the potential health risks of e-cigarettes and raise awareness of the insidious lobbying efforts from the tobacco industry.

## Introduction

### Background

Tobacco control has been identified as a global public health priority by the World Health Organization [1]. Tobacco use is one of the leading causes of preventable deaths globally, and it is responsible for 7 million deaths worldwide [2]. In the United States, the Centers for Disease Control and Prevention (CDC) reported that tobacco use accounted for 1 in 5 deaths, leading to more than 480,000 deaths annually [3]. As such, for tobacco control, understanding public perception of tobacco products and their severe health risks, such as lung cancer, is essential to inform educational campaigns and tobacco control policies.

Particularly, health education campaigns and control policies should pay attention to tobacco consumption trends. In terms of traditional tobacco use, the CDC reported that smoking among adults had declined from 20.9% in 2005 to 13.7% in 2018, and the proportion of smokers who reported quitting had increased [4]. But, the popularity of emerging tobacco products such as electronic cigarettes (e-cigarettes) has been rising steadily since 2010. E-cigarettes are electronic devices that are used to deliver nicotine and other chemicals to users through inhalable aerosols. The US Surgeon General declared that e-cigarette use was an epidemic among youth in 2018, given that 21% of high school seniors in the United States reported using e-cigarettes in the preceding 30 days in the same year [5]. In particular, Juul captured and dominated 73% of the e-cigarette market through its product promotion featuring youth culture and lifestyle on different social media platforms [6].

The overwhelming popularity of e-cigarettes may be due to conflicting messages in the public. Some argue that e-cigarettes could help with smoking cessation as they appear to pose fewer health risks than traditional cigarettes [7-9]. Meanwhile, others cautioned against e-cigarette consumption as they contain nicotine, a highly addictive drug, that can lead to the use of other tobacco products [10-12]. Even though there is no established connection between e-cigarette use and severe diseases such as lung cancer, there is evidence of lung injuries associated with its use [13-15]. Notably, the CDC declared an outbreak of lung injuries associated with e-cigarette use in 2019 [16].

### Related Work

Survey studies have investigated the perceived associations of smoking traditional tobacco products and e-cigarettes with health diseases such as lung cancer. Smoking is recognized as a major risk factor for lung cancer by the public [17]. In contrast, the public may not view e-cigarettes as a likely cause of lung cancer [18,19] but, instead, may view e-cigarettes as a safe alternative to combustible cigarettes [20,21]. There are very few studies that have compared public perceptions of both smoking and vaping in relation to severe health consequences using social media data. Currently, a growing body of social media studies [22,23] on e-cigarettes and smoking show that e-cigarettes are often presented as a positive and healthier alternative to smoking, despite controversies surrounding their effectiveness in smoking cessation and the likelihood of initiating adolescents to consuming other tobacco products. Nevertheless, there is limited evidence about the extent that the public perceives lung cancer to be a health consequence of both smoking and vaping [24-26]. Such comparisons are critical as they can highlight contextual, psychological, and behavioral factors specific to the reasons for which individuals consume different tobacco products. This knowledge is valuable as it can inform strategic messaging and educational programs to facilitate behavioral changes in reducing tobacco consumption [27].

Furthermore, to the best of our knowledge, none of the existing social media studies has investigated spatial patterns of tobacco conversations on social media in relation to implementation of health policies. For example, it has been found that the number of obesity-related policies in certain geographic regions were associated with an increase in obesity prevention discussions on Twitter within the same area [28]. This likely suggests that the number of health policies, or the policy environment in general, have a reciprocal relationship with consumers’ health awareness. Putting this in the context of tobacco control, the number of state-level tobacco policies in the United States may be a reflection of the risk perception of tobacco products and e-cigarettes, and this heightened awareness of risk on a societal level may be impetus for policy makers to enact more laws to rein in tobacco consumption. Therefore, it is crucial to study how tobacco policies of the various states are associated with public perceptions and discussions of health effects of tobacco products on social media.

### Tobacco Discourse During US National Cancer Prevention Month

While the majority of social media studies largely examine public discourse of e-cigarette and smoking in general, this study examined tobacco discourse in the context of US National Cancer Prevention Month, which is an annual campaign led by the American Institute for Cancer Research in the month of February that aims to foster cancer knowledge and promote cancer prevention practices [29]. Particularly, lung cancer, which is a deadly disease that is caused by tobacco consumption [30], is one of prominent cancer topics during the campaign. Past research has shown that month-long cancer campaigns, such as National Cancer Prevention Month, are the key to increasing awareness of cancer and its associated risk factors [31]. As such, National Cancer Prevention Month offer opportunities to examine e-cigarette and smoking discourse related to lung cancer.

Past research has shown that public discourse during cancer campaigns may be different from that during other months. Cancer campaigns raise public awareness by promoting cancer conversations about risk factors and preventions on Twitter [32]. Also, public discourse during cancer campaigns is often driven by health organizations, advocacy groups, and influential personalities such as celebrities [32,33]. The agenda of cancer campaigns typically involves disseminating cancer education messages, advocating prevention engagement, and sharing affective stories of survivors [33,34]. In addition to these messages, Twitter users could respond and selectively follow and express their own opinions on lung cancer. As such, public discourse during campaigns would likely reflect how the public interact with official messages on tobacco consumption and lung cancer. This knowledge can be important for public health officials in identifying gaps in health education.

### Objective

First, we aimed to examine if National Cancer Prevention Month plays a role in promoting conversations on the link of e-cigarettes and smoking with lung cancer. Second, we aimed to examine and compare public discourse in the United States on smoking and e-cigarette in the context of lung cancer on Twitter during National Cancer Prevention Month. Third, we aimed to examine if there were spatial patterns of smoking and e-cigarette’s themes in the United States during National Cancer Prevention Month. Fourth, we explored the relationship between e-cigarette and smoking discussions on Twitter during National Cancer Prevention Month with implementation of tobacco control policies. As such, we put forth 4 research questions: (1) Does national cancer prevention month promote e-cigarette and smoking conversation related to lung cancer? (2) What are the key themes in e-cigarette and smoking tweets within the broader context of lung cancer discussion during National Cancer Prevention Month? (3) Are there geospatial differences in how e-cigarette and smoking tweets were distributed across the United States during National Cancer Prevention Month? (4) What is the relationship between the number of tobacco control policies in states and themes of e-cigarette and smoking tweets?

## Methods

### Data Collection

Data were retrieved from an existing data set of US English-based lung cancer tweets that contained the term “lung cancer” purchased from Twitter. A list of 28 keywords, such as “e-cigarette,” “vape,” and “juul” [35,36], and another list of 21 keywords, such as “cigarette” and “smoking” [37], were developed and used to extract e-cigarette and smoking tweets from the data set, respectively (Table 1). Tweets that contained both sets of keywords were excluded from further analysis to obtain a proper comparison.

**Table 1:** Search keywords for data collection.

Topic	Keywords
E-cigarette	electronic cigarette; vap*; e-cig*; ecig*; e cig; e-pen; epen; e pen; e-juice; ejuice; e juice; e-liquid; eliquid; e liquid; esmoke; e-smoke; e smoke; e-hookah; ehookah; e hookah; e-pipe; epipe; e pipe; atomizer; juul; njoy; v2 cig; joye510
Smoking	cig*; tobacco; waterpipe; water pipe; hooka; smok*; chew; nicotine; shisha; sheesha; bidi; beedi; kretek; narghile; argileh; cheroot; snuff; snus; betel; gutkha; toombak

### Data Processing and Analysis

R statistical software (The R Project) was used for textual analyses. Data were preprocessed and cleaned before advanced textual analysis. Texts were formatted to lower case. Different forms of phrases that had the same meaning were transformed into a common format to facilitate future text processing, such that “e cig,” “e-cig,” “ecig,” and “electronic cigarette” were reformatted into “ecigarette.” Common English stop words, such as “the” and “of,” special characters, and punctuations were removed. The remaining texts were tokenized and lemmatized to further avoid inflected words. The word “lung cancer” was also removed from the analysis.

### Topic Modeling

Topic modeling, using latent Dirichlet allocation, was employed to understand the differences between themes of e-cigarette and smoking in the context of lung cancer discourse. Latent Dirichlet allocation is a popular and widely used algorithm for topic modeling, by which documents are modeled as mixtures over topics and a topic is characterized as a distribution of words [38]. R software (topicmodels package [39]) was used.

The latent Dirichlet allocation algorithm requires a predefined number, *k*, of topics, which we determined with the perplexity and log-likelihood indices. Both indices have been conventionally used to evaluate the model [38,40]. A lower perplexity score and a higher log-likelihood score indicate better generalization performance. We trained topic models from *k*=2 to *k*=20. To maximize the diversity of discussion while at the same time minimize topic overlaps, we selected the *k* model when the *k+1* model did not improve [27,38]. After the topics were generated, we named each topic by examining keywords and posts that were representative of those topics. Topics deemed to have similar themes were combined to facilitate discussions.

To evaluate the prevalence of topics, we used methods described in [27] to determine the topic of each tweet. The output of topic modeling included estimates of the proportion of each topic present in each tweet. Based on this output, we assigned each tweet the topic that had the highest predicted proportion. For example, the tweet “February 7, 2018. The day everyone who juuls simultaneously got lung cancer” was estimated to be 1.1% similar to other tweets under the topic *affective reasoning*, 1.1% similar to other tweets under the topic *personal experiences*, 95.6% similar to other tweets under the topic *sarcasm*, 1.1% similar to other tweets under the topic *cognitive reasoning*, and 1.1% similar to other tweets under the topic *e-cigarette risk* and was, therefore, classified under the topic *sarcasm*.

We compared the temporal distribution of e-cigarette and smoking tweets using chi-square analysis.

### Geospatial Analysis

The geolocation of a tweet was determined by the self-reported location in the profile of the relevant Twitter user. We imported the location strings into the Google Maps geocoordinates application programming interface (API) to obtain the geocoordinates and the corresponding states in the United States. Then, we manually checked to ensure the state information is correct for each tweet. Tweets that did not have a user-reported location or whose reported location string did not return any results were excluded from the geospatial analysis. To further understand the spatial distribution of themes, ratio values were calculated for each state by dividing the number of tweets in each theme by the total number of tweets in each state. We plotted the ratio values by state to visualize the spatial distribution patterns.

To further understand how state policies might affect twitter discussions of e-cigarette and smoking, we compared the number of tweets with the number of state policies (existing, introduced, or recently enacted in the first quarter of 2018) related to e-cigarettes and smoking. State policies were obtained from the tobacco use data portal from the US CDC [41]. For e-cigarettes, the data included policies related to the sale of e-cigarettes to youth, retail licenses to sell e-cigarette, smoke-free indoor, smoke-free on campus, taxes on e-cigarette products, and preemption. For smoking, the data included all policies similar to those for e-cigarettes and additional policies on fire safety. We conducted bivariate correlation analysis (Pearson correlation) of the total number of tweets and the total number of state policies, as well as between the themes of tweets and types of tobacco control policies.

## Results

### Temporal Distribution of E-cigarette and Smoking Tweets

**Figure 1:**
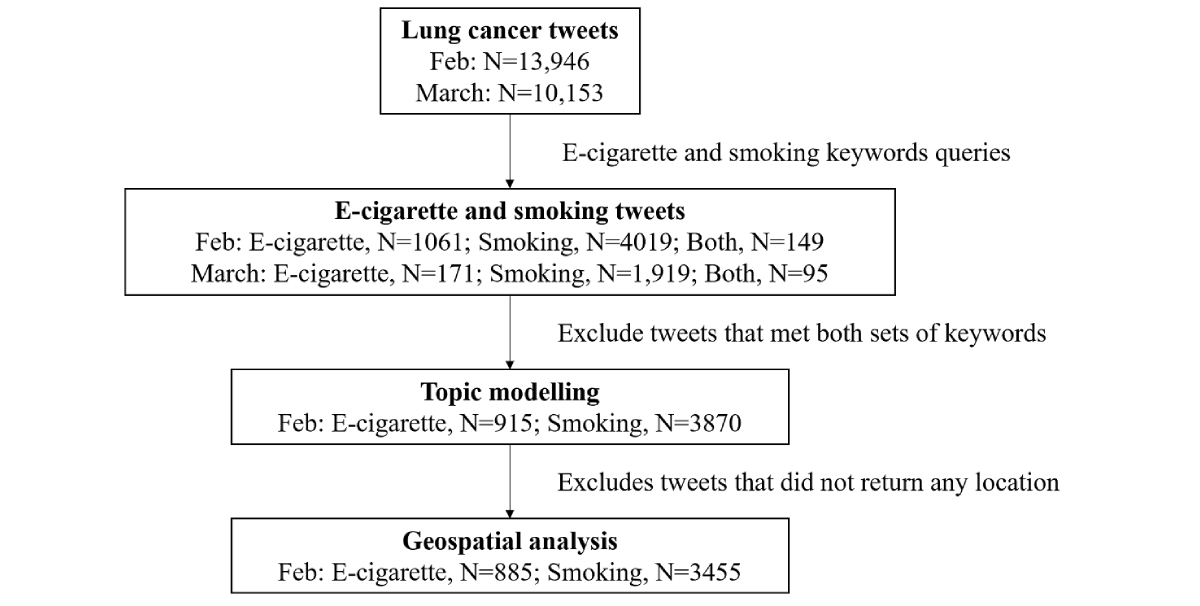
Study flowchart.

The data set had 13,946 publicly available tweets obtained during National Cancer Prevention Month (ie, February) and 10,153 tweets obtained during March in 2018 in the United States. The keyword queries returned 1061 e-cigarette tweets and 4019 smoking tweets during National Cancer Prevention Month, and 171 e-cigarette tweets and 1919 smoking tweets during March (Figure 1). Of these, 149 tweets during National Cancer Prevention Month and 95 tweets during March contained both sets of keywords and were removed. This yielded 6.56% (915/13,946) e-cigarette tweets and 27.7% (3870/13,946) general smoking tweets made by 839 and 3501 unique users during National Cancer Prevention Month, and 0.75% (76/10,153) e-cigarette tweets and 18.0% (1824/10,153) general smoking tweets made by 57 and 1643 unique users during March.

There was a significant difference between the temporal distributions for e-cigarette and smoking tweets (χ^2^=256.85, *P*<.001). This suggests that National Cancer Prevention Month did promote lung cancer discussion associated with e-cigarette and smoking.

### E-cigarette and Smoking Tweets Themes

During National Cancer Prevention Month, 3 major e-cigarette themes—comprising 5 topics—emerged (Table 2). The topics *affective reasoning*, *cognitive reasoning*, and *sarcasm* were categorized under the overarching theme *e-cigarette debate*. First, Twitter users made the link between e-cigarettes and lung cancer based on emotional evaluation of their experiences or anecdotal stories they have heard. Second, there were those who discussed and cognitively processed e-cigarette information presented to them. For instance, one of the tweets highlighted that lung cancer could take years to develop and simply “juuling” for a short period may not be enough to develop lung cancer. Third, some of the Twitter users expressed sarcasm when discussing e-cigarettes, pushing back on the idea of “juuling” and lung cancer. These 3 topics accounted for half of all tweets (454/915, 49.7%). The second major theme was *e-cigarette risks* (145/915, 15.8%), where many tweets discussed scientific evidence on the risks of e-cigarette on lung diseases. The third major theme was *personal experience*s, which constituted approximately one-third of the tweets (316/915, 34.5%). Many tweets in this category encouraged others to stop vaping, with users citing stories they had heard about someone who contracted lung cancer by consuming e-cigarettes.

**Table 2:** E-cigarette themes from tweets (based on latent Dirichlet allocation algorithm).

Theme		Words	Examples	Tweets (n= 915), n (%)
**E-cigarette debates**				454 (49.7)
	Affective reasoning	Juul, hit, kid, people, rumor, cause, shit, really, report, untrue	Drop your juuls like deadass. Have heard of three people my age who have been diagnosed with lung cancer from juuls. Feel like we all saw this coming	158 (17.3)
	Cognitive reasoning	Juul, year, cause, cancer, develop, know, take, use, lung, say	When people think that juuls give you lung cancer but lung cancer takes years to develop	127 (13.9)
	Sarcasm	Juul, everyone, day, February, simultaneously, friend, today, people, college, think	February 7, 2018. The day everyone who juuls simultaneously got lung cancer.	169 (18.5)
Personal experiences		Friend, cousin, stop, girl, good, sister, neighbor, immediately, son, sorority	STOP JUULING IMMEDIATELY‚ My best friends neighbors girl friend’s sorority sister’s cousin’s step son got lung cancer from a single hit of juul. Drop these cancer sticks.	316 (34.5)
E-cigarette risks		Juul, link, lung, kid, disease, ecigarette, severe, flavoring, hit, addict	ecigarette Flavorings linked to Severe LUNG disease https://t.co/2VhStSkI0s #lungdisease #lungcancer #ecigarettes #cancer #ecigaretteflavoring #severlungdisease #howbadareecigarettes	145 (15.8)

Unlike themes expressed in e-cigarette tweets, which showed that users were divided over the association of e-cigarettes with lung cancer, those expressed in smoking tweets (Table 3) showed that users were largely unanimous in perceiving the link between smoking and lung cancer. More than half of tweets (2251/3870, 58.1%) were classified under the theme *policy discussion*, which equated the importance of tobacco control policies with that of gun control policies. Furthermore, more than 20% of tweets (843/3870) focused on the theme *smoking risks*—tweets promoted smoking cessation by mentioning scientific facts of smoking and its relation to lung cancer. Another major theme was *personal experiences*, with 20% of the tweets (776/3870) having stories of how users or their families suffered lung cancer because of smoking.

**Table 3:** Smoking themes (based on latent Dirichlet allocation) from tweets.

Theme			Words		Examples		Tweets (n= 3870), n (%)
**Policy discussion**							2251 (58.1)
	Tobacco lobbying	Tobacco, kid, one, lose, prevent, explain, love, lobbyist, invite, this		This is like inviting tobacco lobbyists to explain to kids who have lost loved ones to lung cancer how we can prevent smoking deaths. Lobbyists don’t deserve a seat at this table.		1831 (47.3)	
	Smoking control policy	Smoker, cigarette, gun, smoke, cause, blame, tobacco, gum, chew, death		Higher prices / taxes on cigarettes=less deaths due to lung cancer. Seat belts / stricter regulations on vehicle safety=less auto deaths		420 (10.8)	
Smoking risks			Smoke, cancer, smoker, lung, non, die, people, risk, quit, cigarette		433 Americans die daily from #lungcancer. The majority of people living with lung cancer r nonsmokers or have quit smoking. Anyone, smoker or nonsmoker, can get lung cancer. While smoking greatly increases the risk of #lungcancer , NO ONE DESERVES CANCER. @theNCI #LCSM https://t.co/9CtilnJLzm		843 (21.8)
Personal experiences			Smoke, get, cigarette, people, die, tobacco, cause, someone, kill, make		Great! My big brother was smoking for the last fifty years Lung cancer finally killed him. Small pain under the arm one sunny morning. Two years later a 3cm tumor killed him.		776 (20.1)

### Spatial Distributions of E-cigarette and Smoking Tweets and Themes

**Figure 2:**
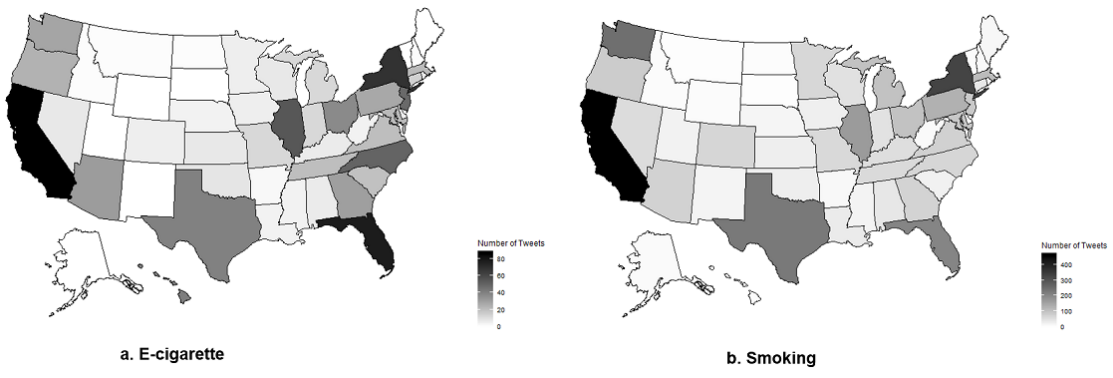
The spatial distribution of e-cigarette and smoking tweets mentioning lung cancer during US national cancer prevention month (February 2018).

The geospatial analysis included 96.7% (885/915) of the e-cigarette tweets and 89.3% (3455/3870) of the smoking tweets during National Cancer Prevention Month. Overall, discussions of e-cigarettes and smoking in relation to lung cancer occurred mostly in the coastal areas and the eastern part of the country (Figure 2). California, New York, Florida, Texas, and Illinois had the most tweets about e-cigarettes and smoking. We focused our analysis on states that had more than 20 tweets in order to draw meaningful distribution patterns (Figures S1 and S2 in Multimedia Appendix 1).

For e-cigarette tweets, California, Arizona, Pennsylvania, Illinois, and Virginia had more tweets debating e-cigarettes than tweets containing the other two themes. Oregon, Texas, Tennessee, and North Carolina had more tweets asking people to stop vaping based on personal stories. Hawaii and the Washington state had more tweets on scientific evidence showing the link between e-cigarettes and lung diseases.

For smoking tweets, most states predominantly had tweets about policy discussions regarding smoke control and how tobacco control policies were important to reign in tobacco companies, equivalent to how gun control policies would restrict gun lobbyists. Nevada and Kentucky had more tweets about the scientific evidence of smoking risks than those about the other two themes.

### Association With State-Level Tobacco Control Policies

The number of tweets under the theme *e-cigarette risks* was positively associated with the number of both indoor arena and on campus smoke-free policies (smoke-free indoor: *r*_49_=0.33, *P*=.02; smoke-free campus: *r*_49_=.32, *P*=.02). Likewise, the number of tweets under the theme *e-cigarette risks* was positively associated with the total number of e-cigarette policies, *r*_49_=.32, *P*=.02). There were no statistically significant associations between the 3 smoking themes and the number of smoking policies in the context of lung cancer (*P*>.13) (Tables S1 and S2 in Multimedia Appendix 2).

## Discussion

### Principal Findings

We examined the prevailing topics and distributions of discussions in Twitter about e-cigarettes and traditional tobacco consumption during the National Cancer Prevention Month in 2018 within the broader context of lung cancer to offer key insights on how the public perceives health risks of both e-cigarettes and smoking and potentially help public health organizations to be more strategic in their messaging and tobacco control efforts by targeting different tobacco products.

First, the findings of temporal distributions of e-cigarette and smoking tweets suggest that National Cancer Prevention Month promoted both e-cigarette and smoking conversations related to lung cancer. What we found notable was that National Cancer Prevention Month promoted e-cigarette conversations more than smoking conversations in the context of lung cancer. Without the cancer campaigns, lung cancer discourse on Twitter were rarely about e-cigarettes (76/10,153, 0.75%) and mostly revolved around the harms of smoking (1824/10,153, 18.0%). This is likely for a few reasons. In February 2018, the American Cancer Society [42] first released an official statement that discouraged youths or young adults from using any tobacco products including e-cigarettes. As such, it might have generated additional attention to the harms of e-cigarettes in relation to lung cancer during National Cancer Prevention Month. Second, 2018 National Youth Tobacco Survey data raised concerns about the vaping epidemic by showing an alarming surge in e-cigarette use among youths from 2017 to 2018; there was a 78% increase in e-cigarette use among high school students and a 48% increase among middle school students [43]. In addition, the nature of National Cancer Prevention Month itself, as a cancer awareness campaign, did indeed promote public debate and concerns about e-cigarettes on Twitter as demonstrated by our data.

Second, the findings of our thematic analysis suggest that Twitter users were aware of the risks of lung cancer from smoking but were split over the potential health effects of vaping. While some of the Twitter users evaluated the link between e-cigarettes and lung cancer based on personal experiences or anecdotal stories they have heard, others processed e-cigarette information in a more cerebral manner and were convinced of the health risks of e-cigarettes. This split in attitude toward e-cigarettes may be the result of mixed communication messaging from public health organizations. For instance, while the CDC has acknowledged the risks of e-cigarettes, particularly for young people due to the presence of nicotine, the long-term health effects of e-cigarettes have been debated [44]. And the National Academies of Sciences, Engineering, and Medicine [10] released a report concluding that e-cigarette use could not be strictly categorized as harmful or beneficial because it would require more long-term studies on the health effects of vaping.

Another significant finding was that themes of political lobbying and policy making were absent from e-cigarette tweets, but not from those about traditional tobacco consumption, during the cancer campaign. When discussing smoking, Twitter users were mindful of the political lobbying by tobacco industries (and equated it to that of gun lobbyists), but this particular theme was missing from e-cigarette tweets. This is crucial, as it suggests that the political lobbying efforts by e-cigarette companies may not be as visible or prominent as those of the traditional tobacco industry. This is a cause for concern. After all, the tobacco industry is very much involved in the e-cigarette industry, as shown by the acquisition of Juul by Altria (formerly known as Philip Morris) for US $12.8 billion in 2018 [45]. Moreover, in recent years, e-cigarette companies have increased efforts in boosting scientific legitimacy in the context of health effects from consumption of their vaping products. For instance, Juul established the JLI Science lab, to fund scientific research on the effects of vaping products—a move that resembled the tobacco industries’ use of research for political lobbying efforts in the 1980s [46]. In terms of policy actions, public debate on the need for tighter regulations over e-cigarettes is critical. Research has shown that supply-side restrictions—such as limiting tobacco retail outlet density—are effective in reducing tobacco consumption [47]. If there are any indications that the current ban on flavored e-cigarettes by the United States and other countries has an effect on the tobacco industry, Altria will revise terms of investments in Juul [48].

Third, this study demonstrated geospatial differences in e-cigarette and smoking discussions on Twitter during National Cancer Prevention Month. In terms of discussing e-cigarette risks, results showed that only 2 states—Hawaii and Washington—had more discussions than those of the others. In the state of Washington, 30% of 12th grade students used e-cigarettes [49], compared to the use of other tobacco products such as smokeless tobacco (4%) or cigars (7%). In the state of Hawaii, high school teenagers vaped twice as much as the national average [50]. To curb the vaping epidemic, Hawaii was one of the first states to raise the legal age of sales for tobacco from 18 years to 21 years, in an effort to prevent young people from nicotine addiction and the harms of tobacco use [51].

There was a positive correlation between discussion of e-cigarette risks and the number of smoke-free policies at the state level. While the findings cannot be used to make any causal claims, it is worth nothing that there may be a reciprocal relationship between public awareness of e-cigarette risks and the passing of smoke-free policies. In other words, when the public becomes aware of the risks of e-cigarettes, they may encourage local representatives to push for more smoke-free policies. At the same time, the passing of smoke-free policies may further increase awareness of e-cigarette risks in the general public.

Though discussion of tobacco risks and the number of smoke-free policies were not correlated, as discussed, people still mentioned the importance of smoking control policies in their tweets. The data suggest that policy engagement and public awareness and discussion of tobacco risks are symbiotic. When the risk awareness of a tobacco product is low, especially for emerging tobacco products such as e-cigarettes, public policy engagement motivated by the community leaders or public health organizations may heighten risk awareness. Once the public are adequately educated on the health risks of a tobacco product (eg, combustible cigarettes), this risk awareness may, in turn, fuel discussions on the need for stringent tobacco control policies, as well as strategically address tactics of political lobbying and messaging by the tobacco industry. In other words, the findings of our study suggest that public health organizations should focus on both improving risk awareness of tobacco products, as well as engaging and educating the public on the importance of tobacco control policies, because these strategies complement and reinforce one another.

We believe that our findings will be useful to help health communication scholars understand public perception and attitudes toward e-cigarettes and smoking. Future studies should (1) test potential reciprocal relationship between policy engagement and risk awareness of tobacco products; (2) investigate the underlying mechanisms, specifically examine how National Cancer Prevention Month or other cancer awareness months could promote e-cigarette discussions with randomized controlled trials, and identify the best strategies in educating the public about the harms of vaping; and (3) replicate our study by examining how cancer awareness months drive conversations about other cancers (eg, breast cancer, prostate cancer) compared with other noncancer awareness months and how various health policies (eg, health insurance) across different states are associated with cancer discussion.

### Limitations

First, while social media sources, such as Twitter, can be used to gauge public opinion and sentiments toward smoking and e-cigarette, we are mindful that they may not be representative, and as such, there are constraints on the generalizability of the results. For example, our data came from publicly available posts, and thus, we were not able to capture themes and sentiments toward smoking and e-cigarettes in private posts. Also, because not all users reported their locations in their profiles, there may be potential selection biases in the geospatial analysis. In addition, we are cognizant that there potentially could be a spill-over effect because our data were collected from consecutive months in February and March. However, we are confident that this was not a major issue of concern given that the number of e-cigarette tweets in February (n=1061) during National Cancer Prevention Month was much greater than the number of e-cigarette tweets in March (n=171). Finally, we excluded tweets with both sets of keywords that might introduce bias and ran the same analyses; we found that the results did not substantially differ; therefore, we are confident that our results are robust.

### Conclusion

The public is aware of smoking and lung cancer risks, but people were generally divided over the risks of e-cigarettes in relation to lung cancer. Public health organizations should invest in strategic messaging efforts over social media to address any misinformation about e-cigarettes because there is a reciprocal relationship between public awareness and discussion on tobacco products on social media and the implementation of tobacco control policies.

## Appendix

Multimedia Appendix 1 The spatial distribution of Twitter themes in relation to lung cancer during US national cancer prevention month (February 2018).

Multimedia Appendix 2 Correlation between the tweet number of themes and the number of state-level tobacco policies.

## Abbreviations

CDC: Centers for Disease Control and Prevention
e-cigarette: electronic cigarette
