# Supplementary material for: Examining Twitter Discourse on Electronic Cigarette and Tobacco Consumption During National Cancer Prevention Month in 2018: Topic Modeling and Geospatial Analysis
Source: J Med Internet Res. 2021 Dec 29;23(12):e28042. doi: 10.2196/28042 (PMC8756341; doi:10.2196/28042)
Supplement: Multimedia Appendix 1 [file jmir_v23i12e28042_app1.docx]

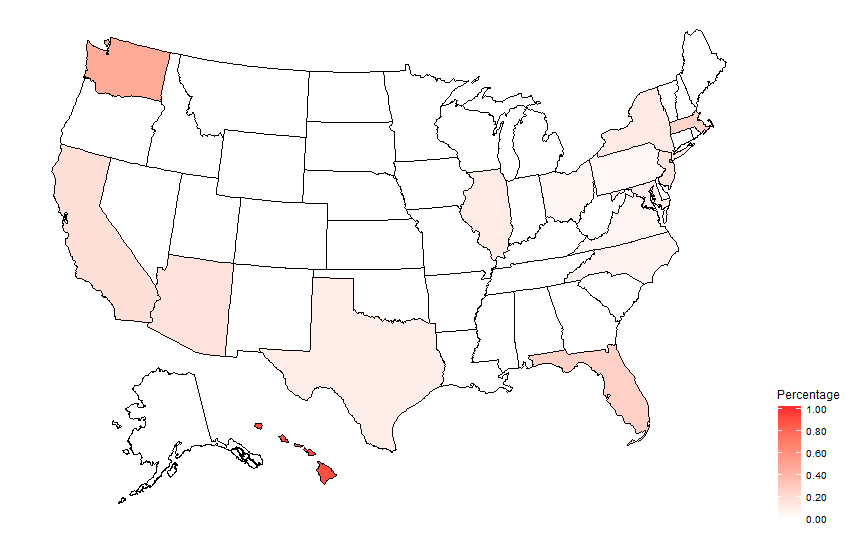

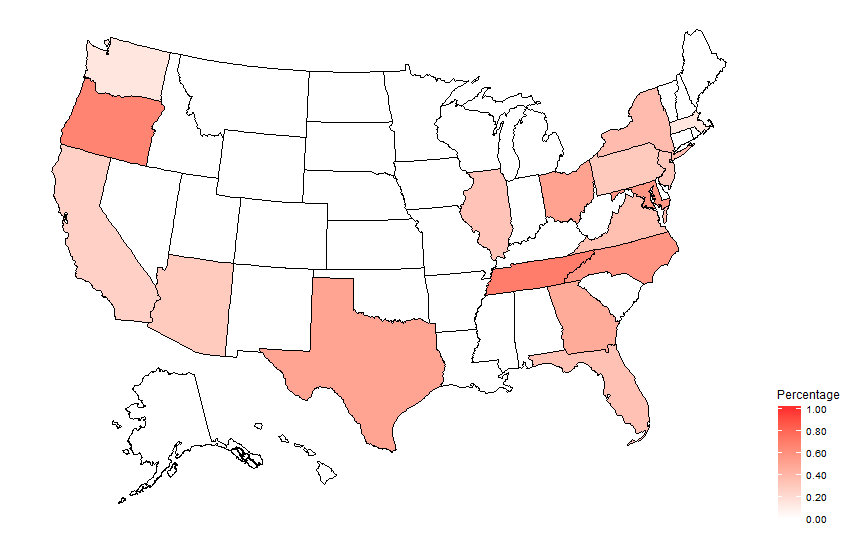

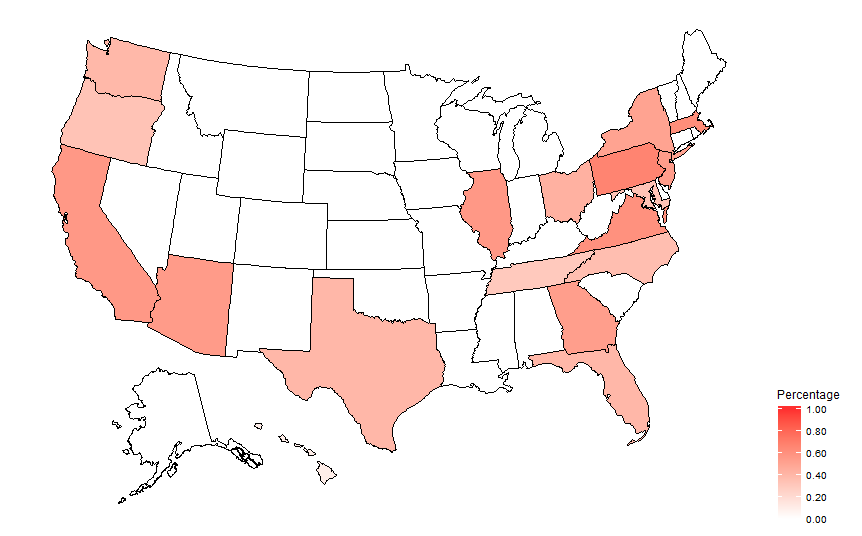


1. Theme 1: E-cigarette debate
2. Theme 2: Experiences from friends
3. Theme 3: E-cigarette as risks

*Figure S1. The spatial distribution of e-cigarette themes in relation to lung cancer during the US national cancer prevention month, February 2018. Only states that had more than 20 tweets were displayed.*


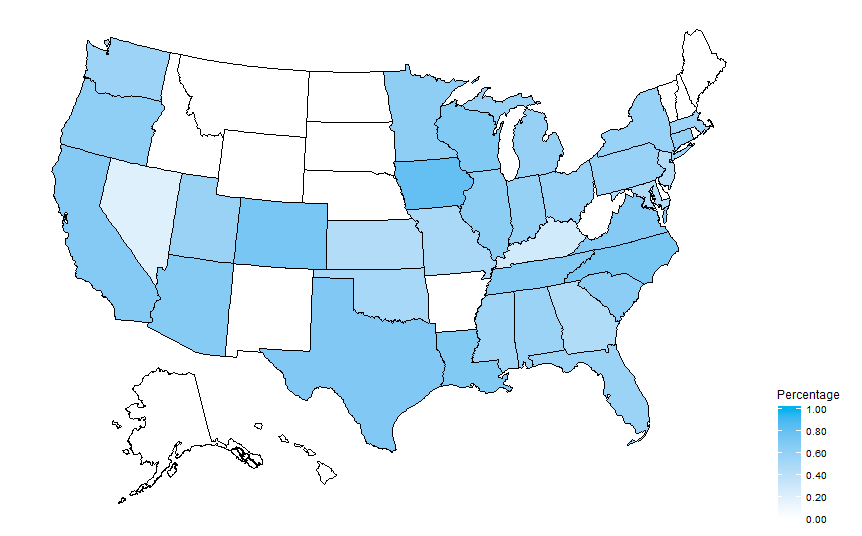

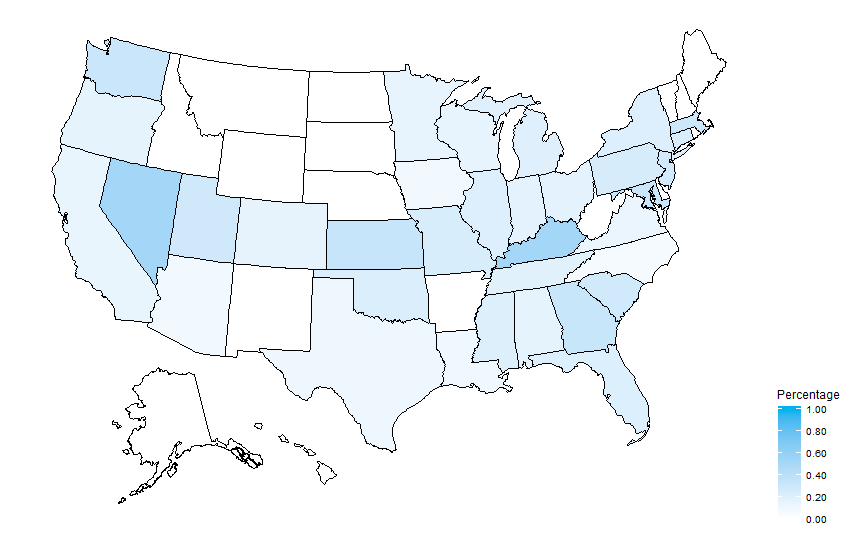

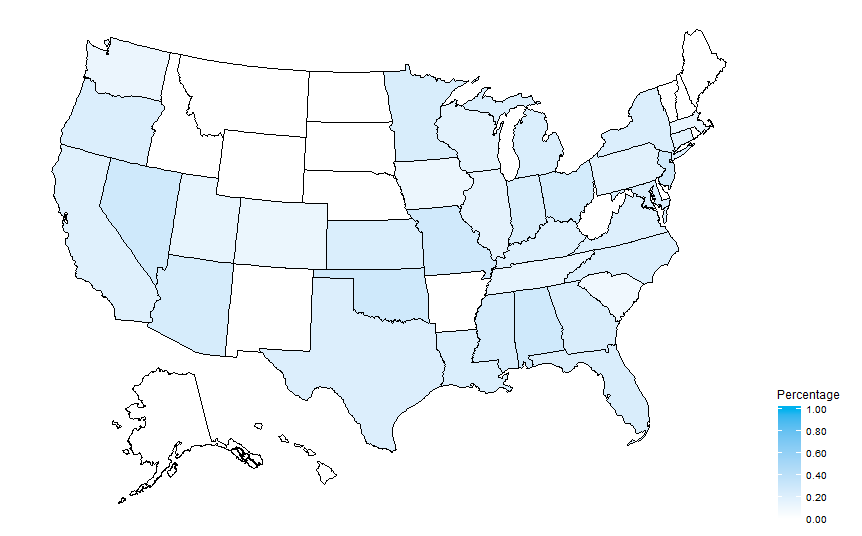


1. Theme 1: Policy discussions
2. Theme 2: Smoking as risks
3. Theme 3: Personal experiences

*Figure S2. The spatial distribution of smoking themes in relation to lung cancer during the US national cancer prevention month, February 2018. Only states that had more than 20 tweets were displayed.*
